# Transcriptome Sequencing of Chemically Induced *Aquilaria sinensis* to Identify Genes Related to Agarwood Formation

**DOI:** 10.1371/journal.pone.0155505

**Published:** 2016-05-16

**Authors:** Wei Ye, Hongqing Wu, Xin He, Lei Wang, Weimin Zhang, Haohua Li, Yunfei Fan, Guohui Tan, Taomei Liu, Xiaoxia Gao

**Affiliations:** 1 State Key Laboratory of Applied Microbiology Southern China, Guangdong Provincial Key Laboratory of Microbial Culture Collection and Application, Guangdong Open Laboratory of Applied Microbiology, Guangdong Institute of Microbiology, Guangzhou, 510070, China; 2 Guangdong Pharmaceutical University, Guangzhou, 510060, China; 3 Inner Mongolia Medical University, Hohhot, 010110, China; University of Western Sydney, AUSTRALIA

## Abstract

**Background:**

Agarwood is a traditional Chinese medicine used as a clinical sedative, carminative, and antiemetic drug. Agarwood is formed in *Aquilaria sinensis* when *A*. *sinensis* trees are threatened by external physical, chemical injury or endophytic fungal irritation. However, the mechanism of agarwood formation via chemical induction remains unclear. In this study, we characterized the transcriptome of different parts of a chemically induced *A*. *sinensis* trunk sample with agarwood. The Illumina sequencing platform was used to identify the genes involved in agarwood formation.

**Methodology/Principal Findings:**

A five-year-old *Aquilaria sinensis* treated by formic acid was selected. The white wood part (B1 sample), the transition part between agarwood and white wood (W2 sample), the agarwood part (J3 sample), and the rotten wood part (F5 sample) were collected for transcriptome sequencing. Accordingly, 54,685,634 clean reads, which were assembled into 83,467 unigenes, were obtained with a Q20 value of 97.5%. A total of 50,565 unigenes were annotated using the Nr, Nt, SWISS-PROT, KEGG, COG, and GO databases. In particular, 171,331,352 unigenes were annotated by various pathways, including the sesquiterpenoid (ko00909) and plant–pathogen interaction (ko03040) pathways. These pathways were related to sesquiterpenoid biosynthesis and defensive responses to chemical stimulation.

**Conclusions/Significance:**

The transcriptome data of the different parts of the chemically induced *A*. *sinensis* trunk provide a rich source of materials for discovering and identifying the genes involved in sesquiterpenoid production and in defensive responses to chemical stimulation. This study is the first to use de novo sequencing and transcriptome assembly for different parts of chemically induced *A*. *sinensis*. Results demonstrate that the sesquiterpenoid biosynthesis pathway and WRKY transcription factor play important roles in agarwood formation via chemical induction. The comparative analysis of the transcriptome data of agarwood and *A*. *sinensis* lays the foundation for elucidating the mechanism of agarwood formation via chemical induction, and thus, enables future improvements in agarwood quality while protecting endangered wild *A*. *sinensi*s.

## Introduction

Agarwood is an aromatic resin-filled wood that is mainly derived from mechanically wounded, chemically induced, or fungal-infected *Aquilaria* spp. trees. *Aquilaria sinensis* is the main plant resource for agarwood in China. Agarwood is a traditional Chinese medicine that is widely used as an analgesic, antitussive, and antiemetic drug [1–4]. However, agarwood production is highly limited because wild *A*. *sinensis* trees are scarce and the rate of agarwood formation from *A*. *sinensis* is low [5]. Consequently, agarwood is very expensive, and its existing supply cannot satisfy the huge demand of consumers. Therefore, elucidating the mechanism of agarwood formation is urgent to lay the foundation for improving agarwood quality and production.

Previous studies have demonstrated that agarwood is formed when *A*. *sinensis* is mechanically injured, chemically induced, or infected by fungi [6–9]. Previous studies were mainly concerning agarwood-inducing methods for *Aquilaria* trees [6–9], few reports were focused on agarwood yield and quality evaluation. Up to now, no standard agarwood quality assessment system is available for the whole agarwood industry. The major components of agarwood are sesquiterpenes and phenylethyl chromones [10,11], and sequiterpene content has been regarded as an important criteria for the evaluation of agarwood quality [10,11]. Higher sesquiterpenes contents were detected in chemically-induced agarwood than those in mechanically wounded agarwood and fungal-infected agarwood [8]. Accordingly, the quality and yield of agarwood induced by chemical methods are higher than those induced by mechanical injury and fungal inoculation [8]. Adding salicylic acid and jasmonic acid methyl ester into *A*. *sinensis* suspension cells can also induce the production of sesquiterpenes, including α-guaiene, α-humulene, and δ-guaiene, which are characteristic components of agarwood [9, 12]. The “agarwood formation induced by defense response” hypothesis indicates that the duct around a wound can transfer inclusions, such as tannin and resin, into the wound. Furthermore, sesquiterpenes and phenylethyl chromones are produced during the defensive response process, which results in the formation of agarwood at the wound location [13].

The transcriptomes of healthy and mechanically wounded *A*. *sinensis* have been sequenced [13]. Accordingly, 30 putatively encoded enzymes involved in the sesquiterpene biosynthesis pathways including mevalonate (MVA) pathway and 1-deoxy-D-xylulose-5-phosphate (DXP) pathway, and transcription factors have been predicted to be related to agarwood formation. Sesquiterpene synthases, HMG-CoA reductase involved in the MVA pathway for the sequiterpene biosynthesis, as well as the WRKY transcription factor have been cloned and expressed in *Escherichia coli*, the functions of which were also clarified [13–18]. It has been reported that sesquiterpene in plant was bio-synthesized by the MVA pathway [16] and the DXP pathway [18]. The two pathways could be divided into three stages including the biosynthesis of isopentenyl pyrophosphate (IPP) and dimethylallyl pyrophosphate (DMAPP), the biosynthesis of direct precursor and the production of terpenoids. [16, 19]. Meanwhile, the sesquitepene content in agarwood from chemically induced *A*.*sinensis* were higher than that of mechanically wounded agarwood, indicating the higher quality of chemically induced agarwood [8]. To date, however, the mechanism of agarwood formation via chemical induction remains unclear.

In the present study, the white wood part (B1 sample), the transition part between agarwood and white wood (W2 sample), the agarwood part (J3 sample), and the rotten wood part (F5 sample) of an *A*. *sinensis* trunk sample were sequenced. The enzyme expression levels involved in the sesquiterpene biosynthesis pathway and the transcription factors related to the defensive response in different parts of the *A*. *sinensis* trunk sample were compared. The results can provide molecular evidence for agarwood formation and elucidate the mechanism of agarwood formation via chemical induction. Consequently, agarwood quality and yield are improved while protecting endangered wild *A*. *sinensis*.

## Results and Discussion

### De novo assembly and analysis

**Fig 1:**
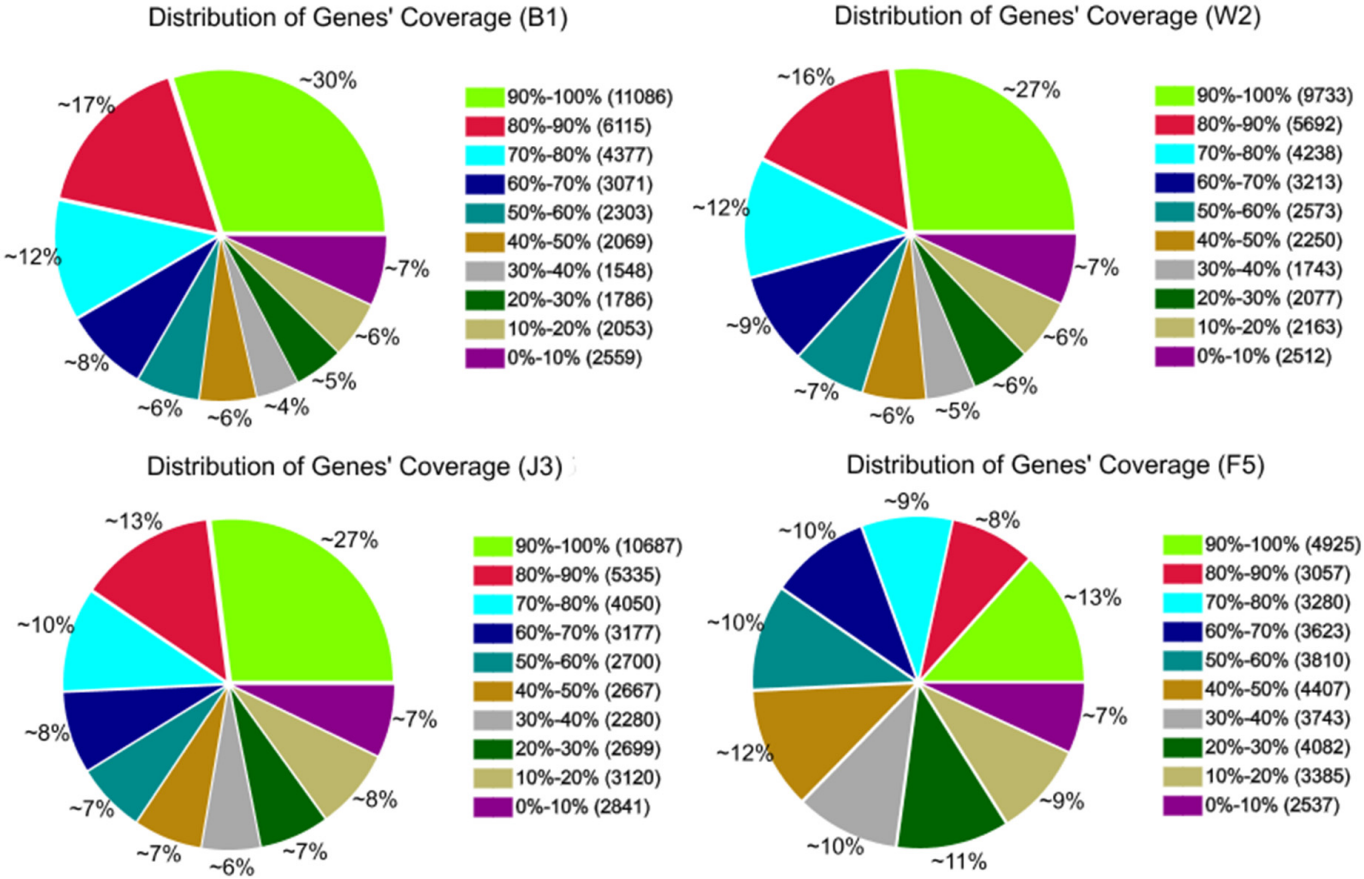
Gene coverage distribution of the sequencing results of different *A*. *sinensis* samples.

A total length of 54,685,634 clean reads was obtained using the Illumina Hiseq^™^ 2000 platform. The total sequence length was 4,921,707,060 nt with a high Q20 value of 97.5%, which indicated the high quality of the transcriptome sequencing. Accordingly, 190,109 contigs with average lengths of 324 were obtained, and 83,467 unigenes with average lengths of 702 nt were assembled [19]. The raw data were deposited in the National Center for Biotechnology Information (NCBI) database with the accession number SRP068230. Fig 1 presents the gene coverage distribution of different samples, including the white wood part of the *A*. *sinensis* trunk (B1), the transition part between white wood and agarwood (W2), the formic acid-induced agarwood part (J3), and the rotten wood part in the *A*. *sinensis* trunk (F5). The significant difference of the gene coverage distributions of B1 and J3 indicated the considerable difference in gene compositions between the agarwood and white wood parts of *A*. *sinensis*.

### Annotation of the assembled unigenes

**Fig 2:**
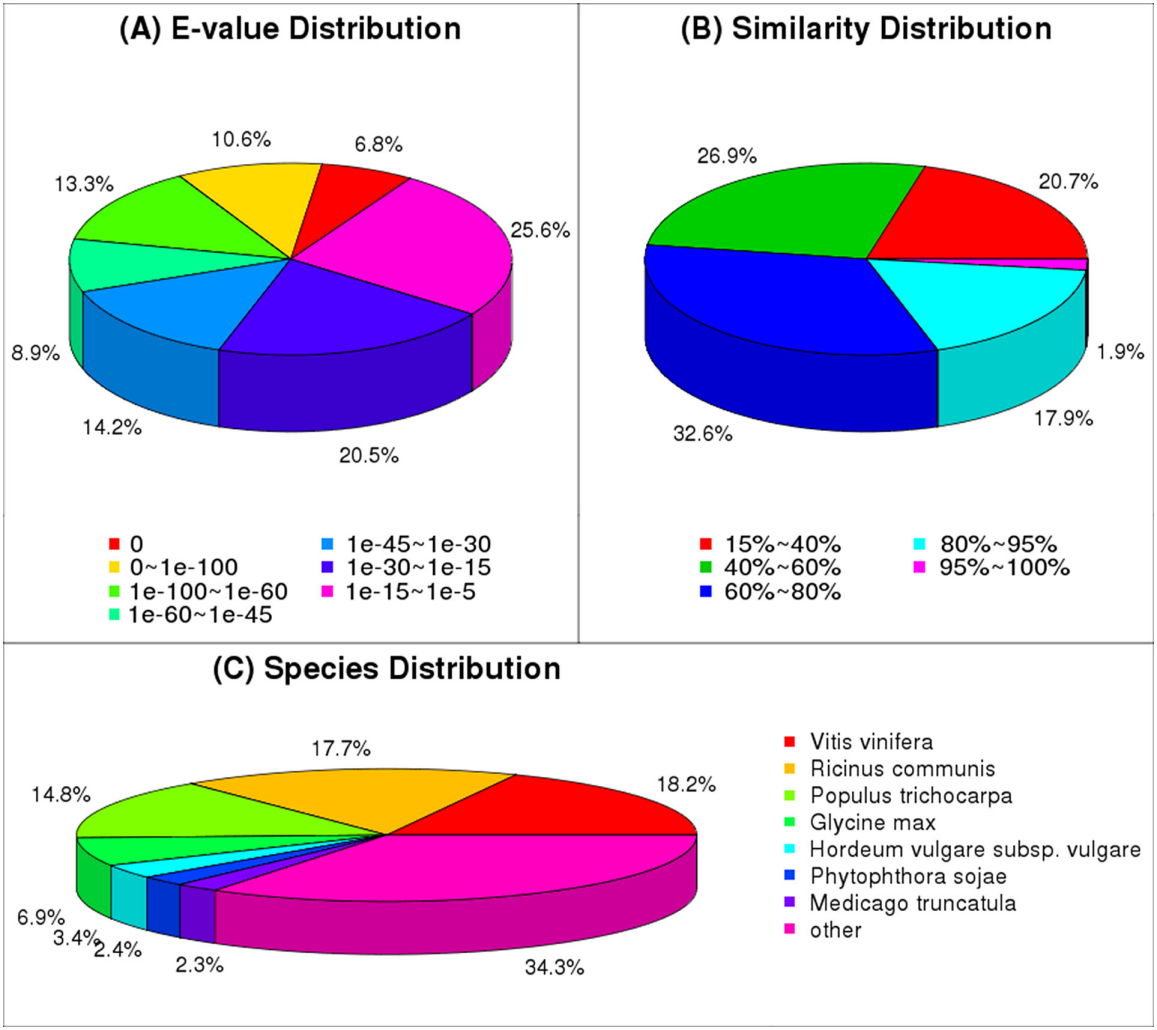
Species statistics and distribution of unigenes in different *A*. *sinensis* samples.

The Nr, Nt, SWISSPROT, KEGG, COG, and GO databases annotated 50,565 unigenes. Nearly 40% of the unigenes remained unannotated. Accordingly, 53.83% of the Nr annotated unigenes exhibited an e-value of <1e^−30^, which implied high identity. Fig 2A illustrates this e-value distribution, whereas Fig 2B illustrates the species distribution of the best hits. The blast results indicated that the genes of five species (i.e., *Vitis vinifera*, *Ricinus communis*, *Populus trichocarpa*, *Glycine max*, and *Hordeum vulgare* subsp. *ulga*) presented the highest similarity with the unigenes of *A*. *sinensis* (Fig 2C).

**Fig 3:**
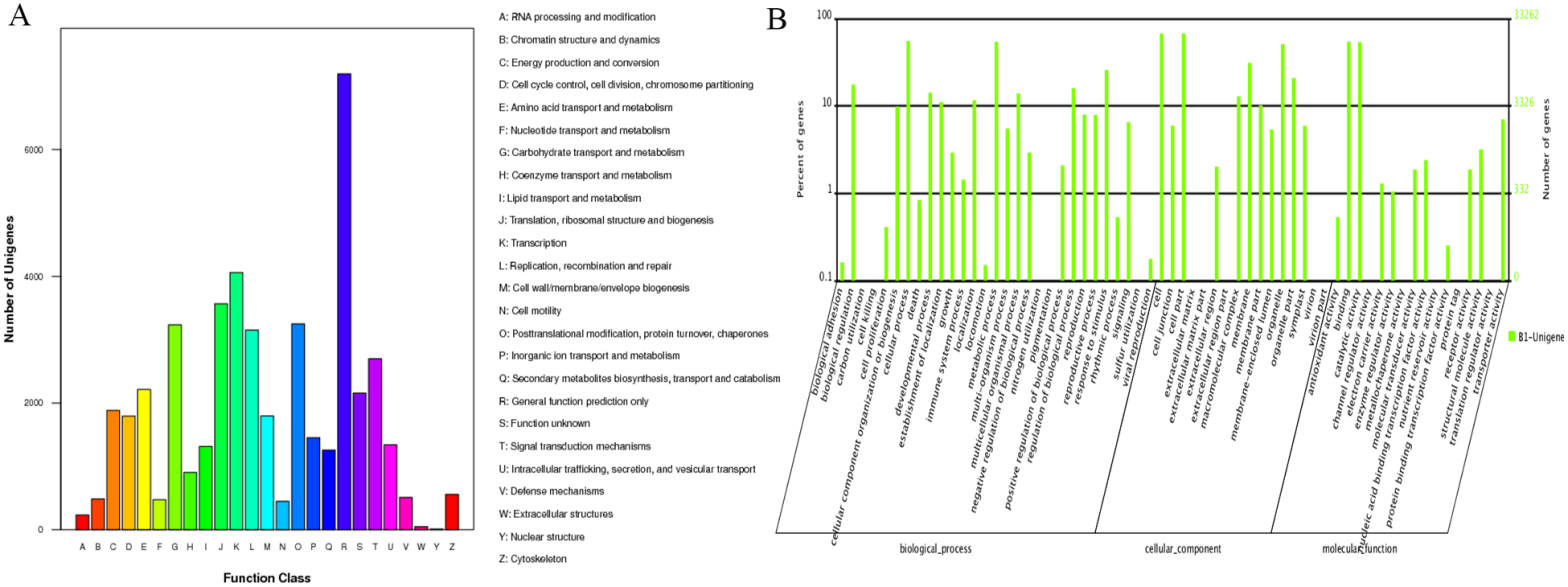
GO and COG function classifications of the unigenes of different *A*. *sinensis* samples.

Subsequently, 23,721 unigenes were annotated and grouped into 25 functional classfications (Fig 3A). The largest cluster was R (general function prediction only), followed by K (transcription). The relatively high frequency of S (function unknown) indicated that many novel genes would still require further identification in *A*. *sinensis* tissues. The GO database annotated 33,262 unigenes. These unigenes were then classified as biological process, cellular component, and molecular functions (Fig 3B). The high percentage of unigenes involved in the binding and catalytic activity functions implied various kinds of secondary metabolites and the complex regulation mechanism in agarwood formation from *A*. *sinensis*. The coding sequences of 49,282 unigenes were predicted from all the blasted unigenes. The lengths of 17.99% amino acids were longer than 300, and 2.92% of which were longer than 1,000.

### Gene differential expression of different parts of the chemically induced *A*. *sinensis* trunk

**Fig 4:**
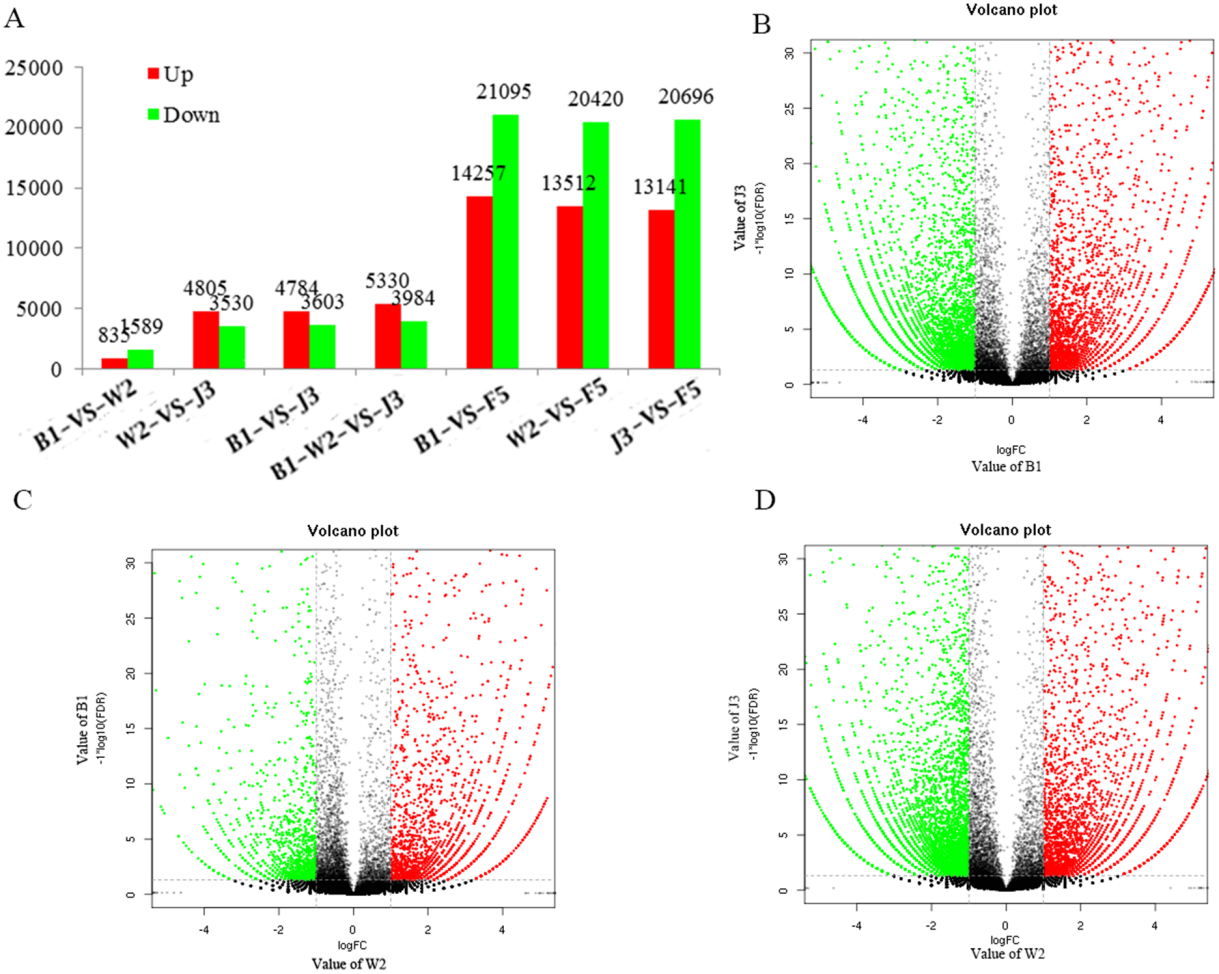
Differential expression of genes in different parts of the *A*. *sinensis* trunk. A) comparison of gene expression levels in different samples, including B1, W2, J3, and F5; B) volcano plot of B1 vs. J3; C) volcano plot of W2 vs. B1; and D) volcano plot of W2 vs. J3.

Fig 4A shows the comparison results of the unigene expression levels in samples B1, W2, J3, and F5 according to the region per million mappable reads (RPKM) value. A comparison with B1 sample showed that the expression levels of 3,603 unigenes in agarwood J3 sample were upregulated, whereas the expression levels of 4,784 unigenes were downregulated. The differential genes between B1 and W2 were considerably less. The expression levels of 835 unigenes were upregulated, whereas those of 1,589 unigenes were downregulated. Over 20,000 unigenes were downregulated, whereas more than 13,000 unigenes were upregulated when the expression level of the unigenes of B1, W2, and J3 were compared with those of the unigenes of F5. This result indicated the significant difference in components between the rotten wood sample infected by fungi and the healthy tissue (white wood) of *A*. *sinensis*. The comparison results implied the incremental changes from the white wood part to the agarwood part. The volcano plots of J3 versus B1, W2 versus B1, and J3 versus W2 showed that considerably more unigenes were upregulated in J3 versus B1 and in J3 versus W2 than in B1 versus W2 (Fig 4B, 4C and 4D). This finding indicated the significant discrepancies in the comparison of unigene expression levels between J3 versus B1 and J3 versus W2. The differences in the unigene expression levels between W2 and B1 were insignificant.

The enriched pathways were obtained after differential expression genes (DEGs) were annotated by the KEGG database. Differentially expressed genes between samples B1 and J3 based on KEGG classification were shown in S1 Fig. Accordingly, 3,590 DEGS were annotated by the KEGG database. The high ratio of the metabolic pathways (30.92%) and the biosynthesis of secondary metabolite pathways (18.3%) in all the DEGs indicated the production of various kinds of metabolites during the agarwood formation process. The high ratio of plant–pathogen interaction pathway (7.44%) implied that unigenes involved in the plant-pathogen interaction pathway also played an important role during the process of agarwood formation by formic acid treatment.

### Sesquiterpenoid biosynthesis pathway

**Fig 5:**
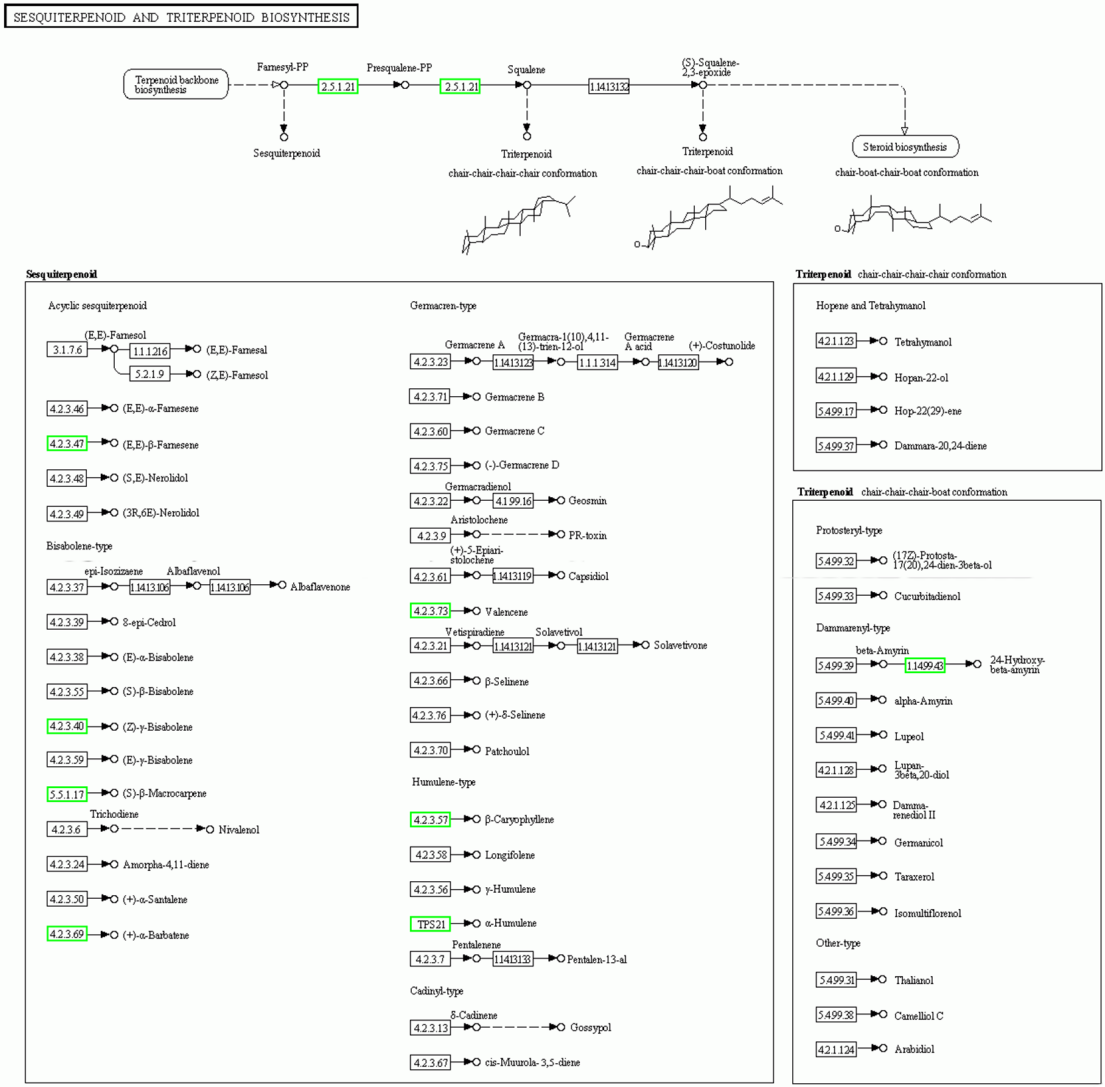
Sesquiterpenoid biosynthesis pathway in *A*. *sinensis*. The green box indicated the expression levels of enzymes involved in the sesquiterpene biosynthesis in the B1 sample were down-regulated compared with those of the J3 sample.

The sesquiterpenes contents in samples B1, W2, J3 and F5 were detected by GC-MS according to the method of Gao [9] (S2 Fig). The types and contents of sesquiterpenes in samples W2 and J3 were listed in S1 Table. The total content of sesquiterpenes was detected in the W2 sample (9.15%), which was much lower than that in the J3 sample (44.32%). No sesquiterpene compound was detected in samples B1 and F5. The detection results of the agarwood obtained using gas chromatography further demonstrated that sesquiterpenes were the main characteristic constituents of agarwood. [10,11] Therefore, the sesquiterpenoid and triterpenoid biosynthesis (ko00909) and the terpenoid backbone biosynthesis (ko00900) pathways (Figs 5 and S3) were annotated.

**Fig 6:**
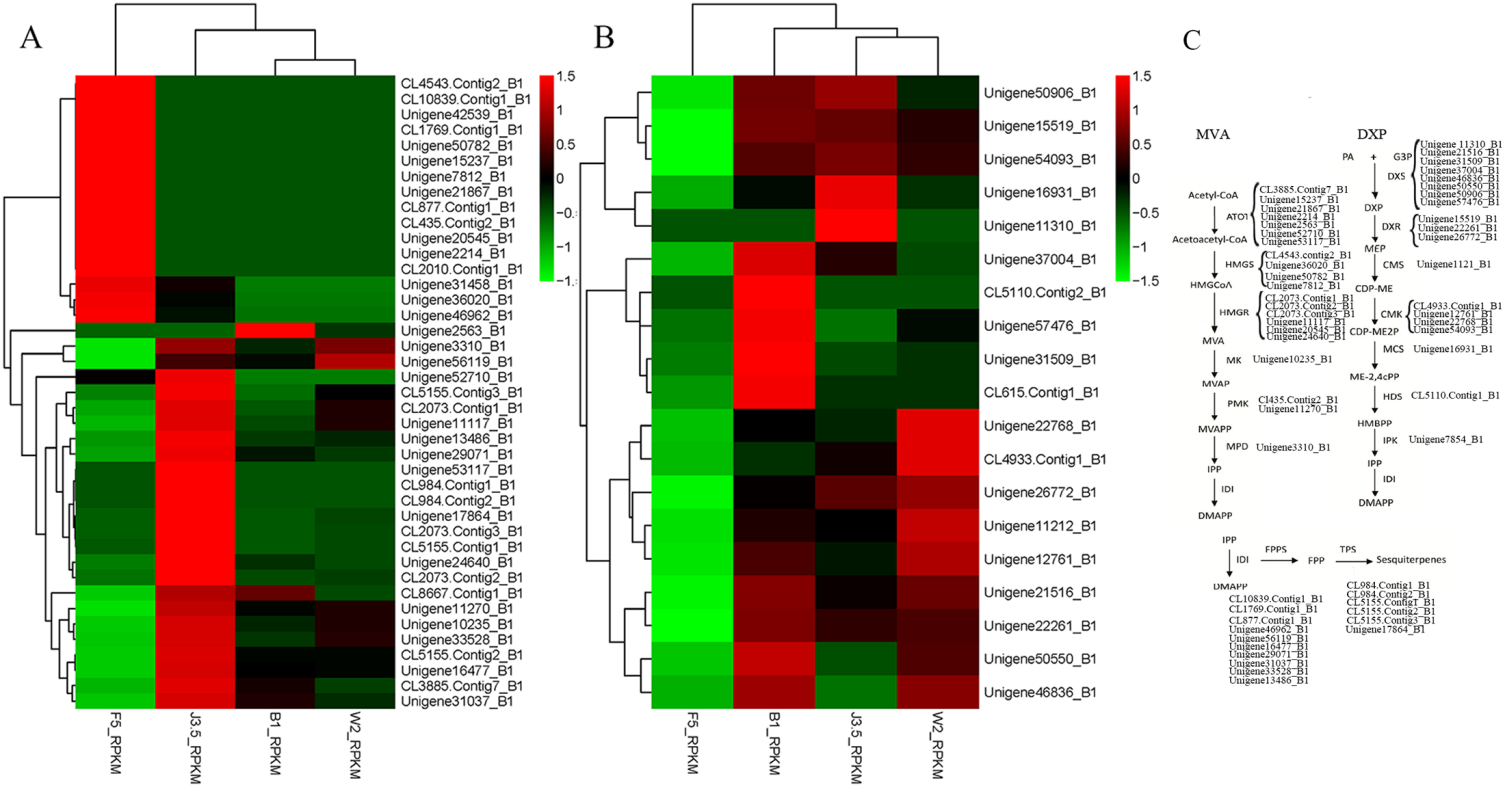
Heat maps of the unigenes involved in the MVA and DXP pathways for sesquiterpene biosynthesis in *A*. *sinensis*. A) heat map of the unigenes involved in the MVA pathway, green color indicated the low expression level of unigenes according to the RPKM value, red color indicated the high expression level. B) heat map of the unigenes involved in the DXP pathway, and C) MVA and DXP pathways for sesquiterpene biosynthesis. Nine kinds of enzymes were involved in the biosynthesis of sesquiterpene by MVA pathway including acetoacetyl-CoA thiolase (ATOT), hydroxymethylglutaryl-CoA synthase (HMGS), hydroxymethylglutaryl-CoA reductase (HMGR), mevalonate kinase (MK), phosphomevalonate kinase (PMK), mevalonate-diphosphate decarboxylase (MPD), isopentenyl diphosphate isomerase (IDI), farnesyl pyrophosphate synthase (FPPS) and sesquiterpene synthase (TPS). There were ten kinds of enzymes involved in the DXP pathway including 1-deoxy-D-xylulose-5-phosphate synthase (DXS), 1-deoxy-D-xylulose-5-phosphate reductoisomerase (DXR), 4-diphosphocytidyl-2C-methyl-D-erythritol kinase (CMK), 4-diphosphocytidyl-2C-methyl-D-erythritol synthase (CMS), 2C-methyl-D-erythritol 2,4-diphosphate synthase (MCS), 1-hydroxy-2-methyl-butenyl 4-diphosphate synthase (HDS), Isoamyl alkenyl monosodium phosphate kinase (IPK), IDI, FPPS and TPS.

The expression levels of the unigene-encoding enzymes, including farnesyl diphosphate and sesquiterpene synthases, involved in the biosynthesis of sesquiterpene by the MVA pathway and DXP pathway were predicted according to the RPKM value. Fig 6 shows the cluster heat maps of the genes involved in the sesquiterpene biosynthesis pathway, including MVA and DXP.

Nine kinds of enzymes and ten kinds of enzymes were involved in the MVA pathway and DXP pathway, respectively [15, 18]. The expression levels of enzymes except ATOT involved in the MVA sesquiterpene biosynthesis pathway reached the maximum value in the J3 sample and exhibited the lowest expression level in the B1 sample. This result implied that the expression levels of these enzymes were probably regulated by the same factor. Furthermore, the prediction results showed that unigenes encoding TPS showed much higher expression level in the J3 sample than other unigenes involved in MVA and DXP pathway in the J3 sample (Fig 6), indicating the important role of TPS gene during the process of agarwood formation by formic acid treatment. Regarding the DXP pathway for sesquiterpenoid production, only the expression levels of enzymes DXS and MCS were significantly higher in agarwood. The enzymes in the MVA pathway presented a significantly higher expression level than those in the DXP pathway. The sesquiterpenes were mainly produced in the cytoplast [15]. The biosynthesis of sesquiterpene by the MVA pathway also occurred in the cytoplast [15], and DXP pathway occurred in plastid [18]. Therefore, the MVA pathway was considered to play a considerably more important role than the DXP pathway in the biosynthesis of sesquiterpenoid during agarwood formation [15,18]. Sesquiterpenes were the main characterized compounds in agarwood. The quality of artificial agarwood was not as ideal as that of natural agarwood because of the relatively lower sesquiterpene content in the former. The sesquiterpene content of natural agarwood was 60%~70%, which was much higher than that of artificial agarwood (8%~50%) [8]. Therefore, elucidating the sesquiterpene pathway in agarwood using genetic engineering approaches will contribute to the improvement of artificial agarwood quality and yield, which will promote the development of the agarwood industry [8–11].

### Differential expression of the transcription factor related to defensive response

**Fig 7:**
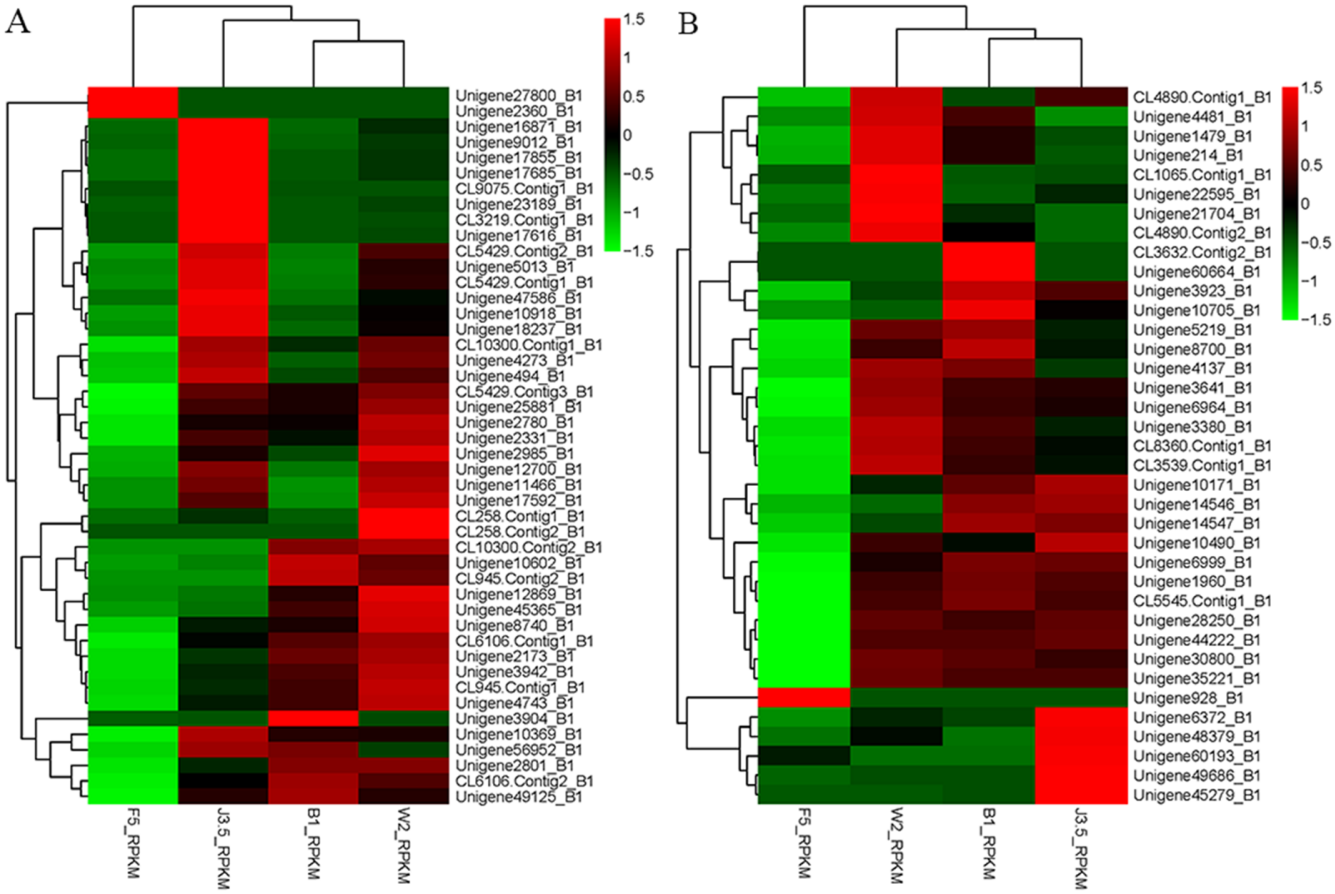
Heat maps of the unigenes involved in the transcription factors WRKY and MYC2 in *A*. *sinensis*. A) WRKY transcription factor; B) MYC2 transcription factor.

The expression of the sesquiterpenoid biosynthetic genes was regulated by the transcription factors WRKY MYC2 and JAZ. The differential expression of the transcription factors WRKY and MYC2 in different *A*. *sinensis* samples was investigated in this study. The cluster heat maps of the genes belonging to the transcription factors WRKY and MYC2 were made according to the RPKM value of the corresponding genes (Fig 7A and 7B). The expression levels of most WRKY transcription factors reached the maximum value in the J3 sample, decreased in the W2 sample, and reached minimum value in the B1 sample (Fig 7A). This result indicated a positive correlation between the expression levels of the WRKY transcription factors and the sesquiterpenoid synthesis gene. Phylogenetic analysis of WRKY transcription factors from chemically-induced *A*. *sinensis* and other species was shown in S4 Fig. The low similarity between WRKY transcription factors from *A*. *sinensis* and other species implied the potential novel WRKY transcription factors in chemically-induced *A*.*sinensis*, indicating the possible different transcription regulatory mechanism in chemically-induced *A*. *sinensis* compared with other species [20–21] The expression level of the transcription factor MYC2 in the J3 sample was only slightly higher than those in samples W2 and B1. MYC2 transcription factor plays an important role in the process of the response to jasmonates stimulation in different plants [22–25]. When plants were stimulated by jasmonates, the SCF protein complex was activated by jasmonates and combined to JAZ protein, leading to the release of MYC2 protein. The released MYC2 protein could further bind to the responsive genes and regulated the physiological process [22–25]. No significant difference was found in the MYC2 gene expression level in different samples indicated that MYC2 transcription factor did not play an important role in the agarwood formation in formic acid-treated *A*. *sinensis*. The rotten wood part also hardly exhibited any WRKY transcription factor expression, which could be attributed to the scarcity of living *A*. *sinensis* cells in the F5 sample. The expression level of the MYC2 transcription factor did not exhibit significant discrepancies in different *A*. *sinensis* samples, suggesting that MYC2 transcription factor did not play an important role in the agarwood formation by formic acid treatment. (Fig 7B).

Transcription factors in plants will mediate defensive response by adjusting the signal of response to the environment. S5 Fig shows the plant–pathogen interaction (ko03040) pathway. WRKY is a type of transcription factor involved in the resistance reaction of different plants. WRKY1 is a positive regulatory factor involved in artemisinin biosynthesis, which binds to the promoter of genes that encode sesquiterpene synthases [18]. The regulatory factors WRKY3 and WRKY6 were related to the biosynthesis of terpenes [20]. Two WRKY genes, namely, NaWRKY3 and NaWRKY6, coordinated responses to herbivory. NaWRKY3 was required for NaWRKY6 elicitation by fatty acid–amino conjugates in *Manduca sexta* larval oral secretions [20]. The two WRKY transcription factors regulated the expression of jasmonic acid biosynthesis genes. The MYC2 transcription factor was mainly involved in the defensive response to jasmonates [24]. Furthermore, the MYC2 transcription factor could only bind to DNA when the plant suffered from jasmonic acid stimulation, which would regulate a series of physiological processes. The expression levels of the sesquiterpenoid biosynthesis genes in *Arabidopis* were upregulated through the interaction of the MYC2 transcription factor with the DELLA protein [22]. The JAZ protein could bind to the MYC2 transcription factor, and thus, block the binding of the MYC2 protein to the promoter. The JAZ protein was also degraded by the 26S proteosome during jasmonic acid stimulation. Therefore, the expression level of the JAZ transcription factor was upregulated during the defensive response process to complement the decreasing JAZ protein [23].

### Quantitative real-time polymerase chain reaction (qRT-PCR) analysis of unigenes

**Fig 8:**
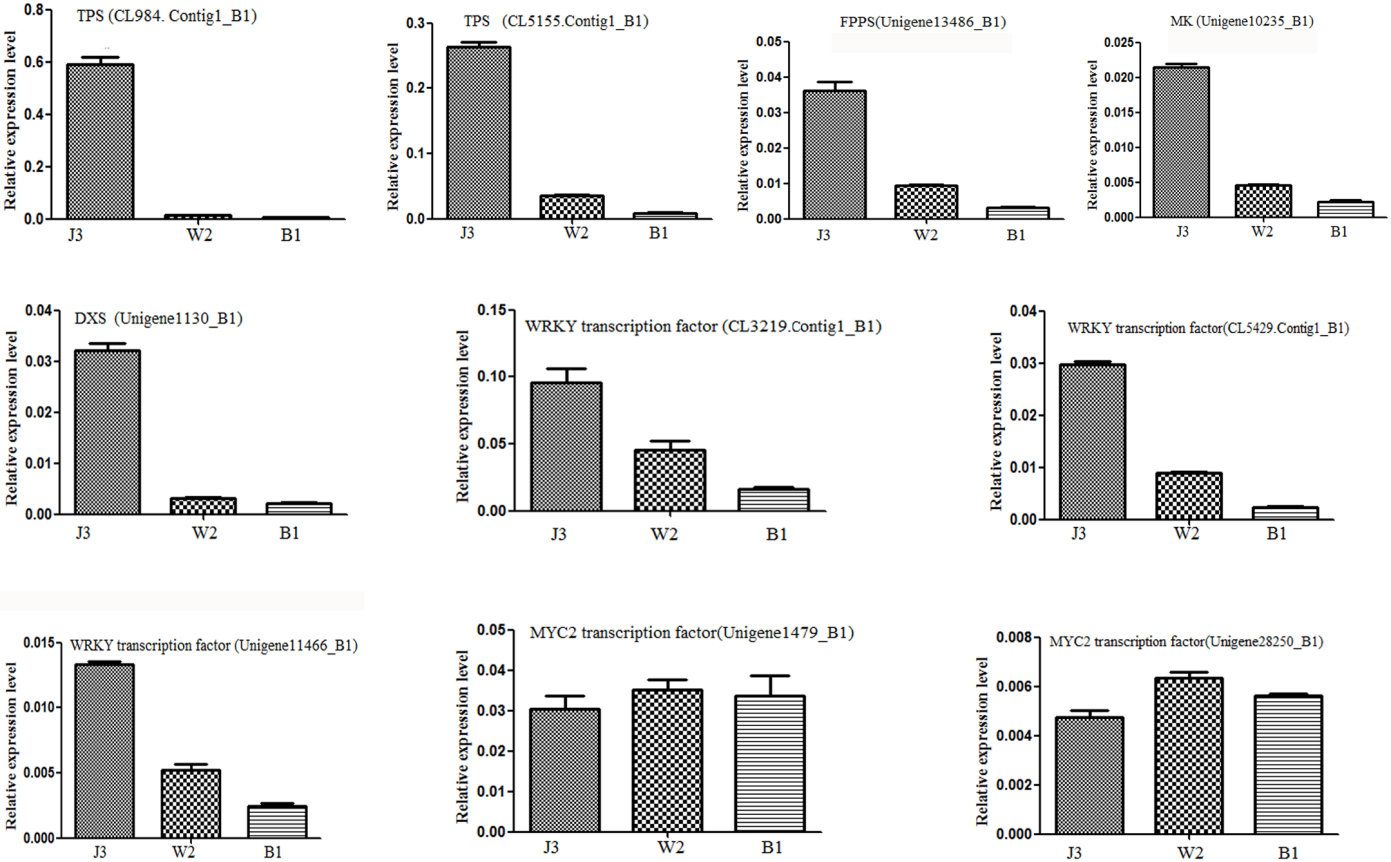
Transcription activities of different genes in *A*. *sinensis* samples identified via qRT-PCR: sesquiterpene synthase (CL984. Contig1_B1, CL5155. Contig1_B1), FPPS (unigene13486_B1), MK (unigene10235_B1), DXS (unigene11310_B1), transcriptional factor WRKY (CL3219.Contig1_B1, CL5429.Contig1_B1 and Unigene11466_B1), and transcriptional factor MYC2 (unigene1479_B1, unigene 28250_B1).

The expression levels of the unigenes involved in sesquiterpenoid biosynthesis and the transcription factors related to defensive responses were validated. Validation was conducted by investigating the expression levels of the key genes for sesquiterpenoid biosynthesis in different samples, including genes that could encode sesquiterpene synthase (CL984. Contig1_B1, CL5155. Contig1_B1), FPPS (unigene13486_B1), MK (unigene10235_B1), DXS (unigene11310_B1), transcriptional factor WRKY (CL3219.Contig1_B1, CL5429.Contig1_B1 and Unigene11466_B1), and transcriptional factor MYC2 (unigene1479_B1, unigene 28250_B1) via qRT-PCR analysis. Fig 8 shows the unigenes CL984. Contig1_B1, CL5155. Contig1_B1, unigene13486_B1, unigene10235_B1 and unigene11310_B1 exhibited the highest expression levels in the J3 sample, considerably lower expression levels in the W2 sample, and extremely low expression levels in the B1 sample, which was in accordance with the prediction by RPKM value (Fig 8). Meanwhile, and the expression level of CL984.Contig1 encoding TPS in the J3 sample was considerably higher than the expression level of CL5155.Contig1 encoding TPS in the J3 sample. The qRT-PCR results indicated that the expression level of the transcription factor WRKY (CL3219.Contig1_B1, CL5429.Contig1_B1 and Unigene11466_B1) in the J3 sample was also higher than that in the B1 sample (Fig 8). However, no significant difference was found in the MYC2 gene (unigene1479_B1 and unigene28250_B1) expression level in different *A*. *sinensis* samples (Fig 8). The results validated the prediction of the cluster heat maps in Figs 6 and 7. This finding demonstrated that the sesquiterpenoid biosynthesis pathway and the transcription factor WRKY that were related to defensive responses played important roles in agarwood formation via chemical induction.

The transcriptome sequencing of healthy and wounded tissues of *A*. *sinensis* was conducted and two cDNA libraries were obtained by Xu [13]. It has been reported that the quality and yield of chemically-induced agarwood was higher than that of mechanically wounded agarwood and fungal-infected agarwood [8]. In this study, the transcriptome of four different parts of formic acid-treated *A*. *sinensis* were sequenced, this is the first report on the transcriptome sequencing of chemically-induced *A*. *sinensis*, more different parts were transcriptome sequenced in our study compared with Xu [13], which could provide more comprehensive molecular basis for the elucidation of agarwood formation mechanism, thus promoting the future development of agarwood industry. Morever, the mechanism of agarwood formation in chemically-induced *A*.*sinensis* in this study could be further investigated by the identification of different sesquiterpene synthases and WRKY transcription factors.

## Conclusion

This study investigates the transcriptome profiles of different parts of a formic acid–treated *A*. *sinensis* trunk sample with agarwood. To the best of our knowledge, this study is the first to use *de novo* sequencing and transcriptome assembly for different parts of a chemically induced *A*. *sinensis*. The sesquiterpene biosynthesis pathway and transcriptional factor WRKY that are related to defensive responses play important roles in agarwood formation via chemical induction. The results of this study provide a molecular proof for the mechanism of agarwood formation induced by chemical agents, and thus, lays a strong foundation for future improvements in agarwood production and quality using chemical and genetic engineering approaches while protecting endangered wild *A*. *sinensis*.

## Materials and Methods

### Inducing agarwood formation in *A*. *sinensis* trunk and definition of the samples

Five year-old matured trees of *A*. *sinensis* were selected randomly from experimental base of Xinyi Rare Agarwood Development Co., Ltd., Xinyi, Guangdong, China (22°21′ N, 110°21′ E, height 119 m). The trees were approximately 3 to 4 m high, larger than 10 cm in diameter, and distances between every two trees were 50 to 70 cm. A drilling device was used to make holes with 0.5 cm in diameter and 4 to 5 cm in depth in the trunks of trees at a height of 1 m. 500 mL formic acid (pH 2.0) was injected slowly into the xylem part of the tree, which can stimulate the tree to produce agarwood [8,26]. The agarwood-inducing experiment was carried out in three trees. Samples were collected with not less than 12 months after agarwood induction. The barks of the *A*. *sinensis* trees were removed. The white wood in the trunk was defined as the B1 sample. The brown agarwood was defined as the J3 sample. The transition part between the B1 and J3 samples with a light brown color was defined as the W2 sample. The rotten wood in the inner area of the trunk was defined as the F5 sample. S6A Fig shows an illustration of the different parts of the agarwood-containing *A*. *sinensis* trunk, and the J3 sample was shown in S6B Fig. Formic acid treatment approach was shown in S6C, S6D and S6E Fig.

### Total RNA extraction and sample preparation for RNA-Seq

The total RNA of samples B1, W2, J3, and F5 was extracted using the modified guanidinium isothiocyanate–cetyltrimethylammonium bromide method. The quantity, purity, and integrity of the extracted RNA were checked on a 1.5% (w/v) agarose gel using a Nanodrop 2000 spectrophotometer. An HPLC Agilent 2100 was used to detect the RIN value of the total RNA. The samples with higher quality (RNA≥6.0) were selected for high-throughput sequencing. The extracted total RNAs amounting to 5.0 μg were resuspended in RNase-free water and stored at −80°C until use. The extracted RNA samples were used for cDNA synthesis. The poly (A) mRNA was isolated using oligo-dT beads (Qiagen). All mRNA was broken into short fragments (200 nt) by adding a fragmentation buffer. First-strand cDNA was generated using random hexamer-primed reverse transcription, followed by the synthesis of the second-strand cDNA using RNase H and DNA polymerase I. The cDNA fragments were purified using a QIAquick PCR extraction kit. The purified fragments were then washed with EB buffer for end preparation poly (A) addition and ligated to sequencing adapters. The cDNA fragments (200 ± 25 bp) were purified and enriched via PCR to construct the final cDNA library following agarose gel electrophoresis and cDNA extraction from gels. The cDNA library was sequenced on the Illumina sequencing platform (Illumina HiSeq™ 2000) using the single-end paired-end (PE) technology within a single run. The original image process to sequences, base calling, and quality value calculation were performed using the Illumina GA Pipeline (version 1.6), in which 90 bp PE reads were obtained [27, 28]. All the sequencing processes of different *A*. *sinensis* samples were conducted in biological triplicate.

### Assembly, comparative analysis, and functional annotation of the transcriptome

Transcriptome sequencing was conducted using the Illumina Hi Seq™ 2000 platform. A PE 100 sequencing strategy was used to assemble all the transcriptomes of different samples. All the sequences were examined to ensure their accuracy. A Perl program was written to select clean reads by removing low-quality sequences (over 50% of the bases had qualities lower than 20 in one sequence), reads with over 5% N bases (bases unknown), and reads with adaptor sequences. The clean reads were then assembled using Trinity to construct unique consensus sequences. Adaptor sequences and low-quality sequences were subsequently trimmed. Short sequences (<50 bp) were removed using a customized Perl program. The obtained high-quality sequences were deposited in the NCBI database and de novo assembled into contigs and transcripts. Transcripts with a minimum length of 200 bp were assembled and clustered using the software CLC NGS Cell under default parameters to reduce data redundancy. The longest sequences in each cluster were reserved and designated as unigenes. Searches were performed using local BLASTX programs against sequences in the NCBI nonredundant (Nr) protein and SWISSPROT databases. The e-value cutoff was 1E^−5^ [29]. Unigenes were tentatively identified according to the top hits against known sequences. The resulting unigenes were used as references to determine the GO and COG terms and were further analyzed using KEGG.

### GO classification and pathway enrichment analyses

DEGs were annotated based on the GO database (http://www.geneontology.org/) using Blast2GO [30] according to their numerical orders in the Nr database to determine the main biological functions. Blast2GO is an all-in-one tool used for the functional annotation of (novel) sequences and the analysis of annotation data, which have been cited by other articles over 150 times. This tool is also a widely recognized GO annotation software. The GO annotations of each DEG were acquired. The WEGO software [31] was then used to obtain the GO functional classifications for all DGEs. The GO enrichment analysis of functional significance terms in the GO database was conducted using a hypergeometric test to find significantly enriched GO terms in DEGs to compare with the genome background.

### Comparative expression analysis

Reads that could be uniquely mapped onto a gene were used to calculate the expression level. The gene expression level was measured by the number of uniquely mapped reads per kilobase of exon RPKM. The RPKM method eliminated the influences of different gene lengths and sequencing discrepancies on calculating gene expression. Therefore, the RPKM value could be directly used to compare the differences in gene expression among the samples. The fold changes of the unigene expression values, with p-values compared with each of the four samples (i.e., B1, W2, J3, and F5), were used to report differential expression. Those with a p-value of <0.05 were considered significant differential expression.

The expression patterns of all the obtained differential expression genes were discovered using the Short Time-series Expression Miner (v1.3.8) [32]. Genes were clustered according to their different expressions in samples B1, W2, J3, and F5. The DGEs that belonged to the same cluster exhibited similar expression patterns with one another. Therefore, clusters with specific expression patterns were selected and verified.

Genes with similar expression patterns typically indicate functional correlation. We performed cluster analysis of gene expression patterns using a clustering software and Java Treeview software [33–34].

### Validation of gene expression

The unigenes related to sesquiterpenoid biosynthesis and defensive responses in different samples were validated via qRT-PCR to verify the quality of sequences assembled in this study. qRT-PCR was performed using a Mastercycler^®^ ep realplex system (Eppendorf, USA) with SYBR Green (Invitrogen, USA) as the fluorescent dye, prepared according to the instructions of the manufacturer. First-strand cDNA was synthesized from 1 μg of total RNA with reverse transcriptase (Takara, Japan) and oligo (dT)_15_ primer. The resulting products were used as templates for qRT-PCR. The primers were designed with Primer Premier 5 according to the unigenes. S2 Table presents a list of the specific primers used for qRT-PCR. The qRT-PCR thermal cycling condition for all reactions was 95°C for 1 min and 50 s, followed by 40 cycles of 95°C for 10 s, 55°C for 33 s, and 68°C for 30 s. All reactions were conducted in biological triplicate. The histone gene was used as the reference gene. The obtained C_T_ values were used as the original data to calculate the relative expression levels of different genes to the histone gene via the 2^−ΔΔCT^ method [34].

## Supporting Information

S1 Fig Differentially expressed genes between samples B1 and J3 based on KEGG classification. (TIF)

S2 Fig GC-MS detection of sesquiterpent contents in different samples. (TIF)

S3 Fig Plant–pathogen interaction pathway map in *A*. *sinensis*. (TIF)

S4 Fig Phylogenetic analysis of WRKY transcription factors from *A*.*sinensis* and other species. (TIF)

S5 Fig Terpenoid biosynthesis pathway in chemically-induced *A*. *sinensis*. (TIF)

S6 Fig Illustration of different parts of chemically induced *A*. *sinensis*. (TIF)

S1 Table Types and contents of sesquiterpenes in samples W2 and J3. (DOC)

S2 Table Primers for the qRT-PCR of unigenes, including CL984. Contig1_B1, CL5155. Contig1_B1, unigene13486_B1, Unigene10235_B1, unigene11310_B1, CL3219. Contig1_B1, CL5429.Contig1_B1, unigene11466_B1, and unigene1479_B1 and unigene28250_B1.
(DOC)

## Abbreviations

ATOT: Acetoacetyl-CoA thiolase
CMK: 4-diphosphocytidyl-2C-methyl-D-erythritol kinase
CMS: 4-diphosphocytidyl-2C-methyl-D-erythritol synthase
DMAPP: dimethylallyl pyrophosphate
DXR: 1-deoxy-D-xylulose-5-phosphate reductoisomerase
DXS: 1-deoxy-D-xylulose-5-phosphate synthase
FPPS: farnesyl pyrophosphate synthase
HDS: 1-hydroxy-2-methyl-butenyl 4-diphosphate synthase
HMGR: hydroxymethylglutaryl-CoA reductase
HMGS: hydroxymethylglutaryl-CoA synthase
IDI: isopentenyl diphosphate isomerase
IPK: Isoamyl alkenyl monosodium phosphate kinase
IPP: isopentenyl pyrophosphate
MCS: 2C-methyl-D-erythritol 2,4-diphosphate synthase
MK: mevalonate kinase
MPD: mevalonate-diphosphate decarboxylase
RPKM: region per million mappable reads
PMK: Phosphomevalonate kinase
TPS: sesquiterpene synthase

## References

[1] Chinese pharmacopoeia Committee (2015): The Pharmacopoeia of People’s Republic of China (I). Beijing: China Medical Science and Technology Press.

[2] VanEtten HD, Mansfield JW, Bailey JA, Farmer EE. (1994) Molecular controls for isoflavonoid biosynthesis in relation to plant and human health. Plant Cell 6: 1191–1192. 12244269 10.1105/tpc.6.9.1191 PMC160512

[3] Ipsen T, Chang YS, Kadir A. (1997). A review on agar (gaharu) producing *Aquilaria* species. J Trop For Prod 2: 272–285.

[4] Pojanagaroon S, Kaewrak C. (2003) Mechanical methods to stimulate aloes wood formation in *Aquiliria crassna* Pierre ex H Lec (kritsana) trees. Acta Hort. (ISHS) 676: 161–166.

[5] Soehartono T, Newton AC. (2000) Conservation and sustainable use of tropical trees in the genus *Aquilaria*. I. Status and distribution in Indonesia. Biol Conse 96: 83–94.

[6] Persoon GA. (2007) Agarwood: the life of a wounded tree. IIAS Newslett. 45: 24–25.

[7] Wei JH, Zhang Z, Yang Y, Meng H, Feng JD, Gan BC. (2010) Production of agarwood in *A* *sinensis* trees via transfusion technique. CN101755629B.

[8] Liu YY, Chen HQ, Yang Y, Zhang Z, Wei JH, Meng H, et al (2013) Whole-tree agarwood-inducing technique: an efficient novel technique for producing high-quality agarwood in cultivated *A*. *sinensis* trees. Molecules 18: 3086–3106. 10.3390/molecules18033086 23470337 PMC6270329

[9] Gao XX, Xie MR, Liu SF, Guo XL, Chen XX, Zhong ZJ, et al (2014) Chromatographic fingerprint analysis of metabolites in natural and artificial agarwood using gas chromatography–mass spectrometry combined with chemometric methods. J Chromatogr B 967: 264–273. 10.1016/j.jchromb.2014.07.039 25129412

[10] Ishihara M, Tsuneya T, Uneyama K. (1993) Fragrant sesquiterpenes from agarwood. Phytochemistry. 33:1147–1155.

[11] Chen HQ, Wei JH, Yang JS, Zhang Z, Yang Y. (2012) Chemical constituents of agarwood originating from the endemic genus *Aquilaria* plants. Chem Biodivers 9:236–250. 10.1002/cbdv.201100077 22344902

[12] Xu YH, Zhang Z, Wang MX, Wei JH, Chen HJ, Gao ZH, et al (2013) Identification of genes related to agarwood formation: transcriptome analysis of healthy and wounded tissues of *A*. *sinensis*. BMC Genomics: 227–242. 10.1186/1471-2164-14-227 23565705 PMC3635961

[13] Gardner RG, Hampton RY. (1999) A highly conserved signal controls degradation of 3-hydroxy-3-methylglutaryl-coenzyme A (HMG-CoA) reductase in eukaryotes. J Biol Chem 274: 31671–31678. 10531376 10.1074/jbc.274.44.31671

[14] Shang CH, Zhu F, Li N, OU-Yang X, Shi L, Zhao MW, et al (2008) Cloning and characterization of a gene encoding HMG-CoA reductase from *Ganoderma lucidum* and its functional identification in yeast. Biosci Biotech Bioch 72:1333–1339. 10.1271/bbb.80011 18460810

[15] Rohmer M (1999) The discovery of a mevalonate independent pathway for isoprenoid biosynthesis in bacteria, algae and higher plants. Nat Prod Rep 16:565–574. 10584331 10.1039/a709175c

[16] Xu YH, Wang JW, Wang S, Wang JY, Chen XY. (2004) Characterization of GaWRKY1, a cotton transcription factor that regulates the sesquiterpene synthase gene (+)-δ-cadinene synthase-A. Plant Physiol. 135:507–515. 15133151 10.1104/pp.104.038612 PMC429402

[17] Ma DM, Pu GB, Lei CY, Ma LQ, Wang HH, Guo YW, et al (2009) Isolation and characterization of AaWRKY1, an Artemisia annua transcription factor that regulates the amorpha-4,11-diene synthase gene, a key gene of artemisinin biosynthesis. Plant Cell Physiol. 50:2146–2161. 10.1093/pcp/pcp149 19880398

[18] Dudareva N, Andersson S, Orlova I, Gatto N, Reichelt M, Rhodes D, et al (2005) The nonmevalonate pathway supports both monoterpene and sesquiterpene formation in snapdragon flowers. Proceedings of the National Academy of Sciences of the United States of America 102: 933–938. 15630092 10.1073/pnas.0407360102 PMC545543

[19] Wu HQ, Wang L, Tao MH, Gao XX, Bai L, Zhang WM. (2013) Transcriptome library construction and sequencing from chemically induced *Aquilaria sinensis*, Biotechnology Bulletin, 2013 (8):63–67.

[20] Skibbe M, Qu N, Galis I, Baldwin IT. (2008) Induced plant defenses in the natural environment: Nicotiana attenuata WRKY3 and WRKY6 coordinate responses to herbivory. The Plant Cell Online 20: 1984–2000. 10.1105/tpc.108.058594 PMC2518244 18641266

[21] Rushton PJ, Somssich IE, Ringler P, Shen QX. (2010) WRKY transcription factors. Trends Plant Sci 15: 247–258. 10.1016/j.tplants.2010.02.006 20304701

[22] Hong GJ, Xue XY, Mao YB, Wang LJ, Chen XY. (2012) *Arabidopsis* MYC2 interacts with DELLA proteins in regulating sesquiterpene synthase gene expression. Plant Cell 24:2635–2648. 10.1105/tpc.112.098749 22669881 PMC3406894

[23] Yu F, Utsumi R. (2009) Diversity, regulation, and genetic manipulation of plant mono- and sesquiterpenoid biosynthesis. Cell Mol Life Sci 66: 3043–3052. 10.1007/s00018-009-0066-7 19547916 PMC11115753

[24] Shin J, Heidrich K, Sanchez-Villarreal A, Parker JE, Davis SJ (2012) Time for coffee represses accumulation of the MYC2 transcription factor to provide time-of-day regulation of jasmonate signaling in Arabidopsis. The Plant Cell 24: 2470–2482. 10.1105/tpc.111.095430 22693280 PMC3406923

[25] Thines B, Katsir L, Melotto M, Niu YJ, Mandaokar A, Liu GH, et al (2007) JAZ repressor proteins are targets of the SCFCOI1 complex during jasmonate signalling. Nature 448: 661–665. 17637677 10.1038/nature05960

[26] Wang L, Zhang WM, Gao XX, Wu GQ, Li HH, Chen Q. (2011) An artificial induction methods of *Aquilaria sinensis*. China Patent No. 201110184345.3.

[27] Li P, Deng WQ, Li TH, Song B, Shen YH. (2013) Illumina-based *de novo* transcriptome sequencing and analysis of *Amanita exitialis* basidiocarps, Gene 532: 63–71. 10.1016/j.gene.2013.09.014 24050899

[28] Gao J, Yu X, Ma F, Li J. (2014) RNA-Seq analysis of transcriptome and glucosinolate metabolism in seeds and sprouts of Broccoli (*Brassica oleracea* var. *italica*). PLoS ONE 9: e88804 10.1371/journal.pone.0088804 24586398 PMC3937326

[29] Wu JK, Zhang WQ, Huang SB, He ZQ, Cheng YB, Wang J. (2013) SOAP fusion: a robust and effective computational fusion discovery tool for RNA-seq reads. Bioinformatics 29: 8. 24123671 10.1093/bioinformatics/btt522

[30] Conesa A, Gotz S, Garcia JM, Terol J, Talon M, Robles M. (2005) Blast2GO: a universal tool for annotation, visualization and analysis in functional genomics research. Bioinformatics 21: 3674–3676. 16081474 10.1093/bioinformatics/bti610

[31] Ye J, Fang L, Zheng H, Zhang Y, Chen J, Zhang Z, et al (2006) WEGO: a web tool for plotting GO annotations. Nucleic Acids Res. 34: 293–297. 10.1093/nar/gkl031 PMC1538768 16845012

[32] Ernst J, Joseph ZB. (2006) STEM: a tool for the analysis of short time series gene expression data. BMC Bioinformatics 7: 191 16597342 10.1186/1471-2105-7-191 PMC1456994

[33] Saldanha AJ. (2004) Java Treeview—extensible visualization of microarray data. Bioinformatics 20: 3246–3248. 15180930 10.1093/bioinformatics/bth349

[34] Huang L, Li GY, Mo ZL, Xiao P, Li J, Huang J. (2015) *De novo* assembly of the Japanese flounder (*Paralichthys olivaceus*) spleen transcriptome to identify putative genes involved in immunity. PLoS ONE 10: e0117642 10.1371/journal.pone.0117642 25723398 PMC4344349

