# Supplementary figures and images for: Transcriptome Sequencing of Chemically Induced Aquilaria sinensis to Identify Genes Related to Agarwood Formation
Source: PLoS One. 2016 May 16;11(5):e0155505. doi: 10.1371/journal.pone.0155505 (PMC4868263; doi:10.1371/journal.pone.0155505)

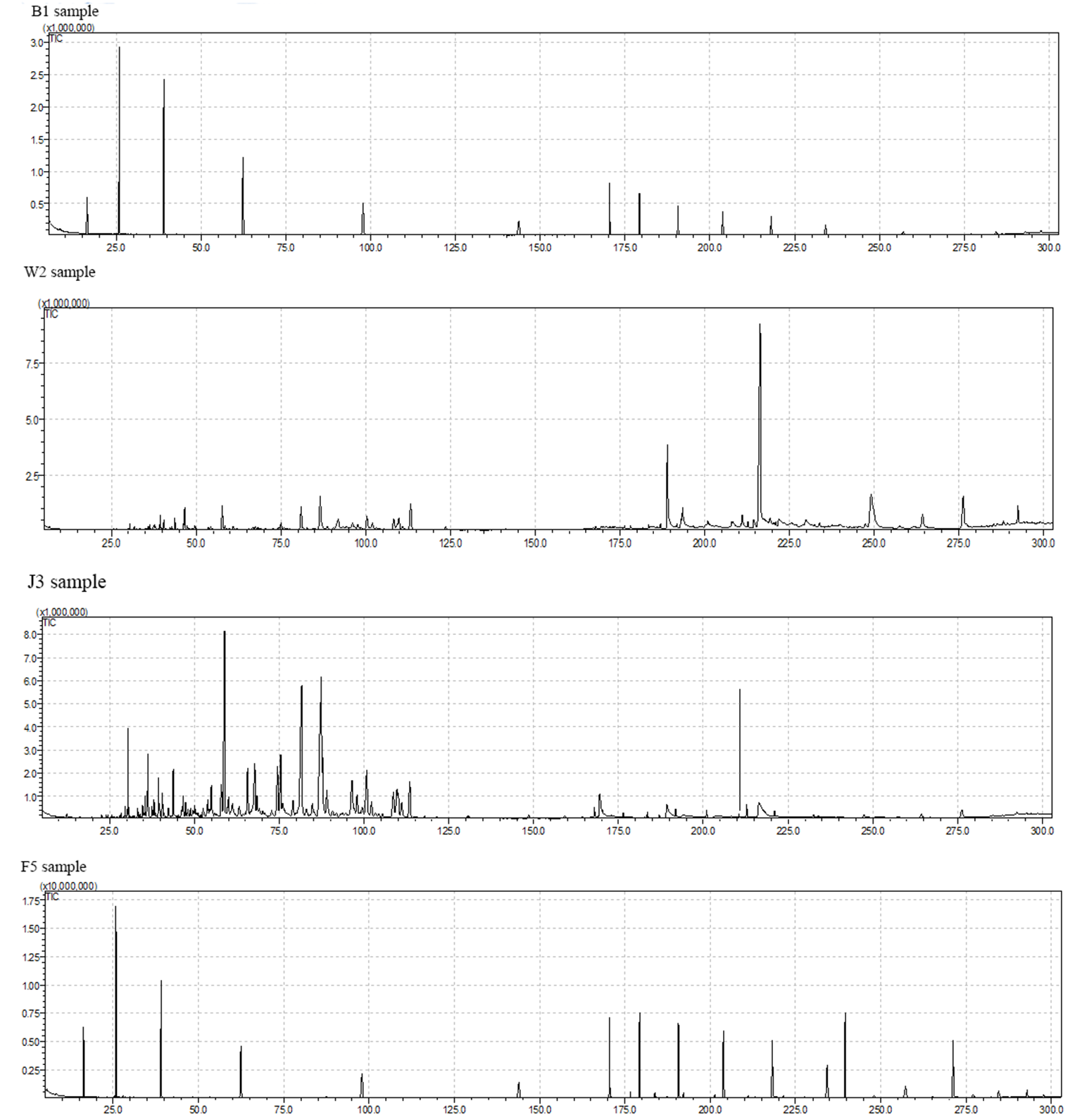

Supplement: S2 Fig — (TIF) [file pone.0155505.s002.tif]

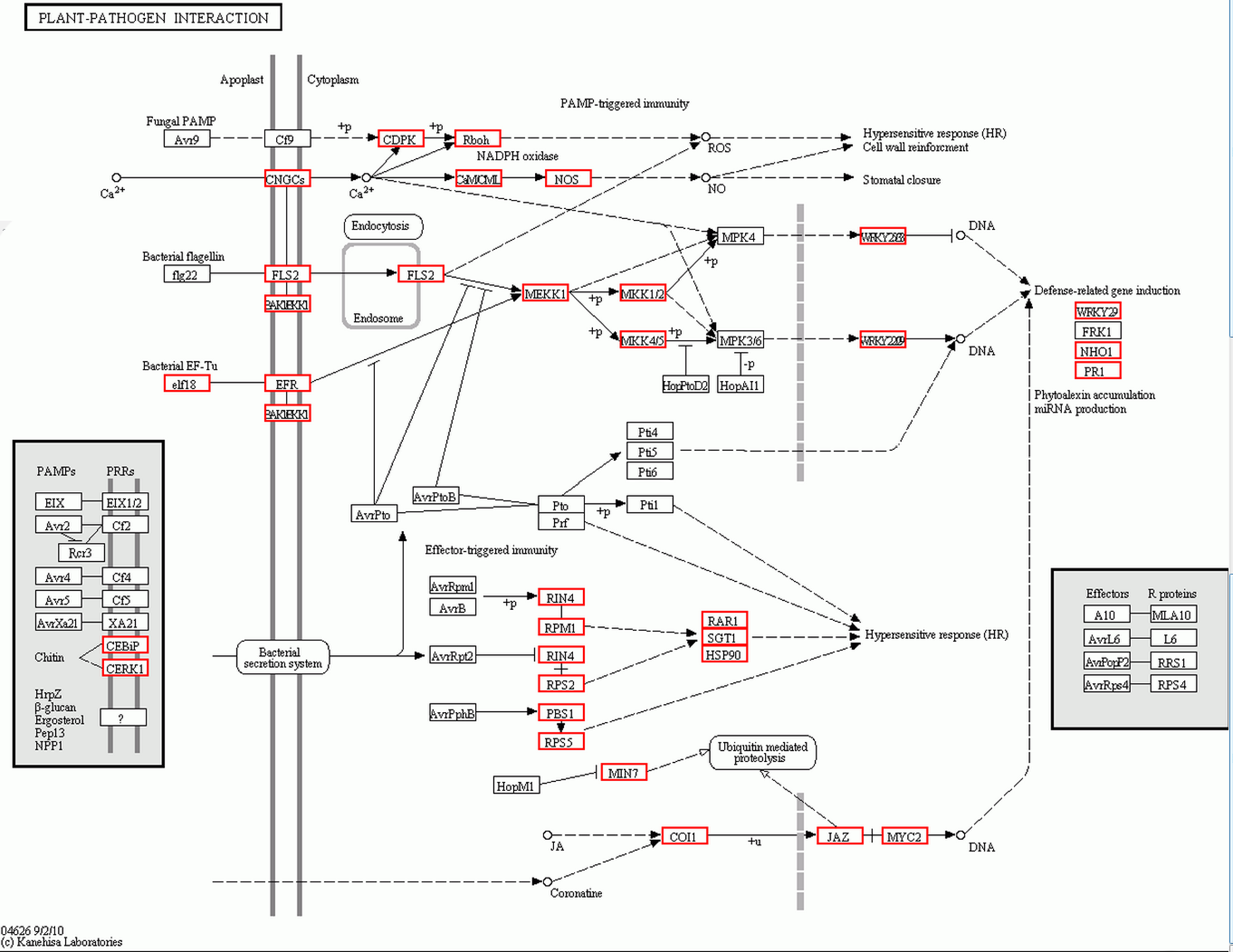

Supplement: S3 Fig — (TIF) [file pone.0155505.s003.tif]

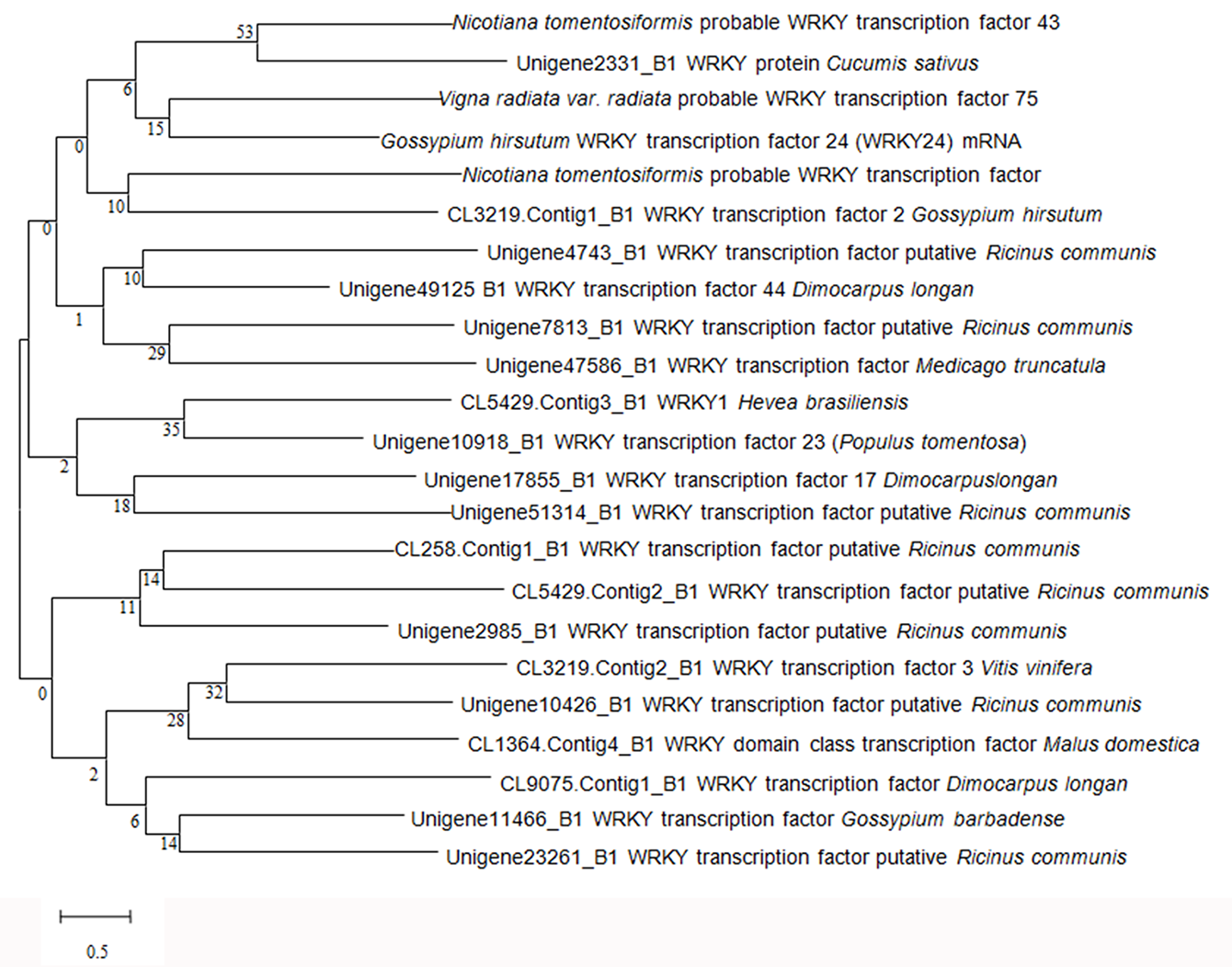

Supplement: S4 Fig — (TIF) [file pone.0155505.s004.tif]

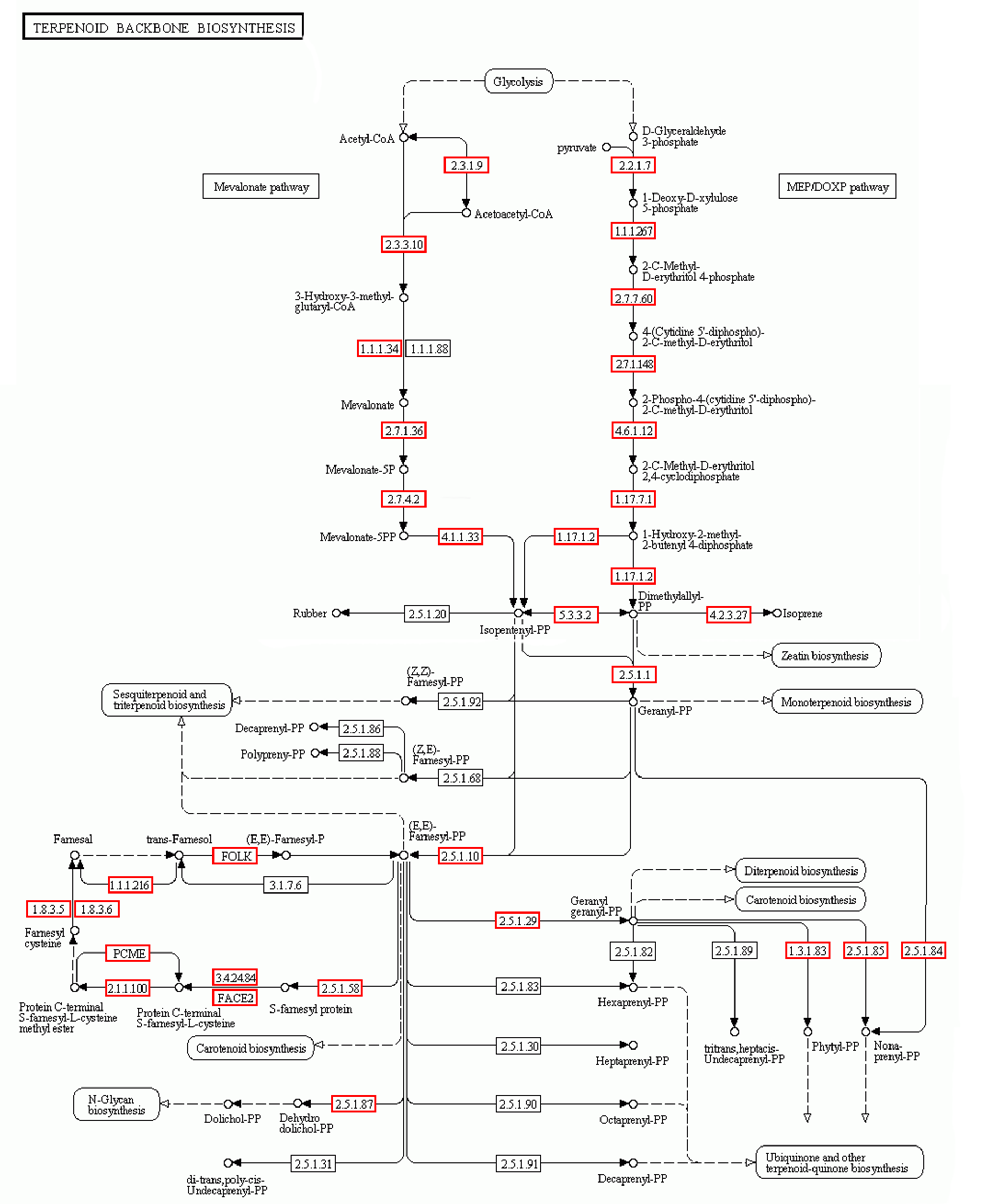

Supplement: S5 Fig — (TIF) [file pone.0155505.s005.tif]

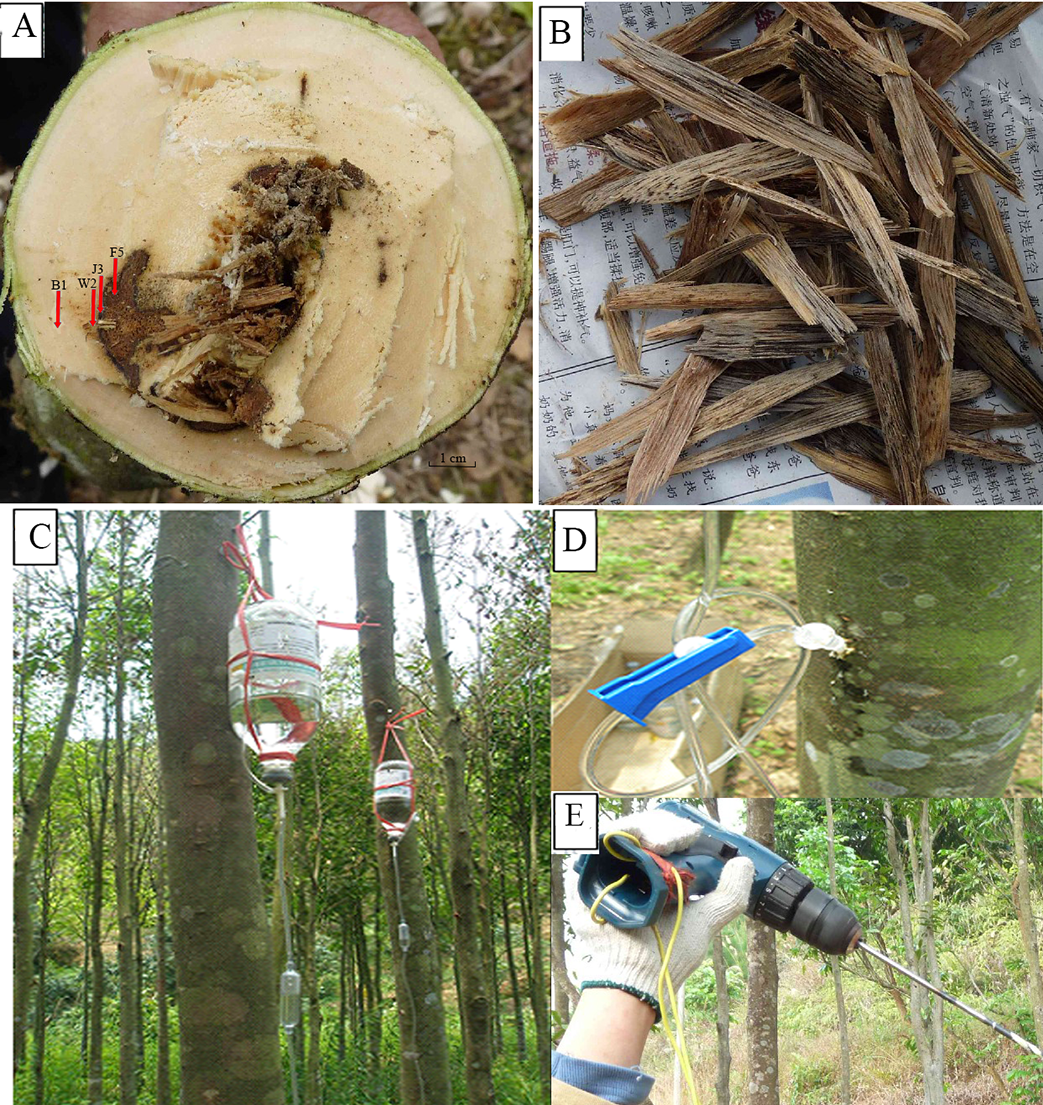

Supplement: S6 Fig — (TIF) [file pone.0155505.s006.tif]
