# Supplementary material for: Transcriptome Sequencing of Chemically Induced Aquilaria sinensis to Identify Genes Related to Agarwood Formation
Source: PLoS One. 2016 May 16;11(5):e0155505. doi: 10.1371/journal.pone.0155505 (PMC4868263; doi:10.1371/journal.pone.0155505)
Supplement: S1 Table — (DOC) [file pone.0155505.s007.doc]

Table 2 Types and contents of sesquiterpenes in samples W2 and J3

| Retention time (min) | Sesquiterpens | Content(%) in  J3 sample | Content(area%) in W2 sample |
| --- | --- | --- | --- |
| 30.395 | Isoaromadendrene epoxide | 1.34 | 0.11 |
| 36.216 | Guaiol | 1.56 | 0.15 |
| 39.381 | Aristolone | 0.91 | 0.46 |
| 43.72 | Longifolenaldehyde | 1.28 | 0.49 |
| 58.749 | Baimuxinal | 8.02 | 0.2 |
| 65.675 | Santalol | 2.09 | 0.21 |
| 74.482 | Cedrane-8,13-diol | 2.83 | 0.09 |
| 75.412 | Vellerdiol | 3.02 | 0.42 |
| 81.589 | Verrucarol | 9.01 | 1.99 |
| 87.058 | Velleral | 11.11 | 3.5 |
| 100.791 | 6-(1-Hydroxymethylvinyl)-4,8a-dimethyl  -3,5,6,7,8,8a-hexahydro-1H-naphthalen-2-one | 3.15 | 1.53 |
