# Supplementary material for: Transcriptome Sequencing of Chemically Induced Aquilaria sinensis to Identify Genes Related to Agarwood Formation
Source: PLoS One. 2016 May 16;11(5):e0155505. doi: 10.1371/journal.pone.0155505 (PMC4868263; doi:10.1371/journal.pone.0155505)
Supplement: S2 Table — Contig1_B1, CL5155. Contig1_B1, unigene13486_B1, Unigene10235_B1, unigene11310_B1, CL3219. Contig1_B1, CL5429.Contig1_B1, unigene11466_B1, and unigene1479_B1 and unigene28250_B1. (DOC) [file pone.0155505.s008.doc]

| Primers | Sequences (5’-3’) |
| --- | --- |
| CL984. Contig1_B1 forward primer | GTTCAGCCAAAACTATACACCTACAT |
| CL984. Contig1_B1 reverse primer | TCTGCTTATATCGTCTCCTATCCCA |
| CL5155. Contig1_B1 forward primer | ATGGTGGAAGGATCTTGATTTCAAAACA |
| CL5155. Contig1_B1 reverse primer  Unigene13486_B1 forward primer  Unigene13486_B1 reverse primer  Unigene10235_B1 forward primer  Unigene10235_B1 reverse primer  Unigene11310_B1 forward primer  Unigene11310_B1 reverse primer | ACCTCTCAATTGCATCTGTGAGTAGCTT  GGGGTGGCGGAGCGCGACG  ATGGCTGCTGTGAGTCTTGG  CCTTGGCTCATTTCCATTAG  TTACGTCCATCTTCGTTTCC  GCGGCAGGGAGTGTTTTCCAC  AAGCCCAGTCTTCTGAGCG |
| CL3219. Contig1_B1 forward primer | GGCTCCTCGAGGAGCTCAACCGGGTC |
| CL3219. Contig1_B1 reverse primer  CL5429.Contig1_B1 forward primer  CL5429.Contig1_B1 reverse primer  Unigene11466_B1 forward primer  Unigene11466_B1 reverse primer | TCTGTTGGATGGCCGCCGGTGACTCC  GATGGGGCAGGGGTGGGTGTG  GATGAAGGGAAGCAGCAGCAG  GAAAGGAGTAGCGTAAATTTGC  AATTACCAAATGTTTTTCCC |
| Unigene 1479_B1 forward primer | TGGGGCCAGTAGAAACACTTAG |
| Unigene 1479_B1 reverse primer  Unigene28250_B1 forward primer  Unigene28250_B1 reverse primer | TCTCTCTCCACTCCAGACTCTC  AATTCCAACTGCAGCTCCTATA  CGGAAGTTGAGCTGGTACGCC |
| Histone forward primer | GTACCGCTACCGGAGGGAAGTTGAAGA |
| Histone reverse primer | CTTCTTGGGCGACTTGGTAGCCTTGGT |

Table S1 Primers for qRT-PCR using cDNAs from different *Aquilaria sinensis* samples
